# Sphingosine-1-phosphate lyase (SGPL1) deficiency is associated with mitochondrial dysfunction

**DOI:** 10.1016/j.jsbmb.2020.105730

**Published:** 2020-09

**Authors:** A. Maharaj, J. Williams, T. Bradshaw, T. Güran, D. Braslavsky, J. Casas, L.F. Chan, L.A. Metherell, R. Prasad

**Affiliations:** a Centre for Endocrinology, William Harvey Research Institute, John Vane Science Centre, Queen Mary, University of London, Charterhouse Square, London, United Kingdom; b Marmara University, School of Medicine, Department of Paediatric Endocrinology and Diabetes, Istanbul, Turkey; c Centro de Investigaciones Endocrinológicas “Dr. Cesar Bergadá” (CEDIE) – CONICET – FEI – División de Endocrinología, Hospital de Niños “Ricardo Gutiérrez”, Buenos Aires, Argentina; d Research Unit on BioActive Molecules (RUBAM), Department of Biomedicinal Chemistry, IQAC-CSIC, Jordi Girona 18-26, Barcelona, Spain

**Keywords:** Sphingolipid metabolism, Mitochondrial dynamics, Steroidogenic capacity, Oxidative phosphorylation, Sphingosine-1-phosphate lyase

## Abstract

Deficiency in Sphingosine-1-phosphate lyase (S1P lyase) is associated with a multi-systemic disorder incorporating primary adrenal insufficiency (PAI), steroid resistant nephrotic syndrome and neurological dysfunction. Accumulation of sphingolipid intermediates, as seen with loss of function mutations in *SGPL1,* has been implicated in mitochondrial dysregulation, including alterations in mitochondrial membrane potentials and initiation of mitochondrial apoptosis. For the first time, we investigate the impact of S1P lyase deficiency on mitochondrial morphology and function using patient-derived human dermal fibroblasts and CRISPR engineered *SGPL1*-knockout HeLa cells. Reduced cortisol output in response to progesterone stimulation was observed in two patient dermal fibroblast cell lines. Mass spectrometric analysis of patient dermal fibroblasts revealed significantly elevated levels of sphingosine-1-phosphate, sphingosine, ceramide species and sphingomyelin when compared to control. Total mitochondrial volume was reduced in both S1P lyase deficient patient and HeLa cell lines. Mitochondrial dynamics and parameters of oxidative phosphorylation were altered when compared to matched controls, though differentially across the cell lines. Mitochondrial dysfunction may represent a major event in the pathogenesis of this disease, associated with severity of phenotype.

## Highlights

• Sphingosine-1-phosphate (S1P) lyase deficiency leads to accumulation of upstream sphingolipid species.
• Acute steroidogenesis is impaired in S1P lyase deficient patient dermal fibroblasts.
• S1P lyase deficiency affects mitochondrial morphology and dynamics.
• Parameters of oxidative phosphorylation are altered in S1P lyase deficiency.

## 1 Introduction

**Fig. 1:**
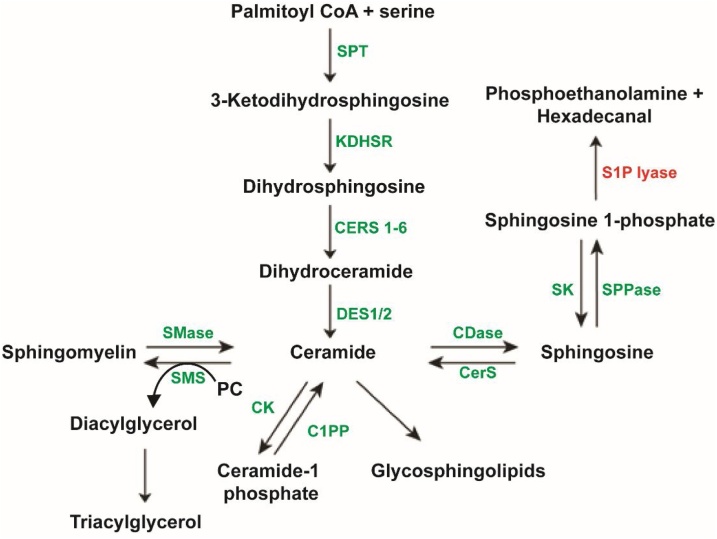
**Final degradative sphingolipid pathway.** Abbreviations: SPT – serine palmitoyl transferase, KDHSR – ketodihydrosphingosine reductase, CERS – ceramide synthase, DES – dihydroceramide desaturase, SMase – sphingomylinease, SMS – sphingomyelin synthase, PC – phosphatidylcholine, CK – ceramide kinase, C1PP – ceramide 1-phosphate phosphatase, CDase – Ceramidase, SK – sphingosine kinase, SPPase – sphingosine 1-phosphate phosphatase, S1P lyase – sphingosine-1-phosphate lyase.

Sphingolipids are ubiquitous structural components of mammalian plasma membranes whose roles have been implicated in signal transduction and cellular recognition pathways [1]. Sphingolipid synthesis involves a series of tightly regulated, enzyme-catalysed steps that initiate in the endoplasmic reticulum from non-sphingolipid precursors progressing to biosynthesis of higher order complex glycosphingolipids within the Golgi apparatus. Despite the diversity within the biosynthetic pathway, sphingolipid metabolism can be said to begin with a common entry point and exit via a single degradative pathway (Fig. 1).

S1P lyase (SGPL1) is a pyridoxal 5’-phosphate-dependent aldehyde-lyase (encoded by the gene *SGPL1*) which binds sphingosine 1-phosphate (S1P) and catalyses the final and irreversible degradative step in sphingolipid metabolism forming phosphoethanolamine and the long chain aldehyde, hexadecanal (Fig. 1). Up until this point in the pathway enzyme reactions are reversible, thus S1P lyase is a major modulator of total cellular sphingolipid intermediates and S1P signalling. Sphingosine 1-phosphate acts as a ligand for a family of five specific G-protein coupled receptors (S1PR1–5) linked to downstream cellular signalling pathways involving angiogenesis, immune cell trafficking, stem cell differentiation and programmed cell death [2]. Under normal physiological conditions, S1P is largely pro-proliferative, suppressing the pro-apoptotic actions of ceramide, however loss of function mutations in *SGPL1* result in a pathological accumulation of S1P which studies have shown is associated with induction of apoptosis [3]. In 2017, Prasad et al. [4], Lovric et al. [5] and Janecke et al. [6], described a series of patients with loss of function mutations in *SGPL1* and a novel syndrome involving primary adrenal insufficiency and steroid resistant nephrotic syndrome. Since the initial publications several case reports have emerged, in total 35 genetically confirmed cases have been published involving 20 kindreds and 20 different mutations in *SGPL1*, only one of which, R222Q, has been described in more than one kindred [[4], [5], [6], [7], [8], [9], [10], [11], [12]]. This disorder is often lethal, with 16 deaths reported among the 35 published cases (in addition to 4 intrauterine deaths). Extra adrenal manifestations including primary hypothyroidism, primary gonadal failure, ichthyosis, neurodevelopmental delay, neurologic abnormalities, lymphopenia and dyslipidaemia have been noted, but were not present universally in affected patients and can vary even within kindreds. Mass spectrometric analysis of sphingolipid intermediates in plasma and conditioned media from patient-derived human dermal fibroblasts revealed elevated ceramide and S1P levels when compared to matched controls suggesting that the underlying pathology in these patients may be due to accumulation of specific sphingolipid intermediates [4,5].

Several sphingolipid intermediates such as ceramide, sphingosine and sphingosine 1-phosphate are known to act *in vitro* as modulators of the steroidogenic pathway, as second messengers, altering downstream expression of steroid responsive transcriptional elements [6]. Furthermore, ACTH promotes the catabolism of sphingosine and ceramide as well as acute activation of sphingosine kinase activity leading to a preferential increase in S1P levels. S1P then acts at multiple levels in the steroidogenic pathway to upregulate cortisol biosynthesis [13]. Acute activation of S1P signalling in H295R (human adrenocortical tumour) cells is postulated to lead to increased transcription of steroidogenic acute regulatory protein (*STAR*) and Hormone sensitive lipase (*HSL*) culminating in an increase in cortisol production [14,15].

Elevations in ceramide levels within mitochondria cause inner mitochondrial membrane dysfunction and since the first and last steps of cortisol biosynthesis occur in this location, induction of mitochondrial dysfunction in S1P lyase deficiency may represent a major event in pathogenesis of the disease.

Therefore, this study investigates the impact of S1P lyase deficiency on mitochondrial morphology and function using patient-derived dermal fibroblasts and a CRISPR-Cas9 generated, *SGPL1*- knockout cell line (HeLa) using a combination of fluorescence microscopy, extracellular flux analysis and mRNA/protein quantification of relevant mediators of respiratory chain function. In addition, as the skin expresses the same genes and proteins involved in the synthesis of adrenal corticosteroids [[16], [17], [18]], use of dermal fibroblasts present an opportunity to study the concomitant impact on steroidogenesis in patient- derived cells.

## 2 Materials and methods

### 2.1 Antibodies and fluorescent probes

Rabbit anti-PARP antibody (Cell Signalling Technologies, 9542S, RRID:AB_2160739), Total OXPHOS Rodent WB Antibody Cocktail (ab110413, RRID:AB_2629281), Purified Mouse Anti-DLP1 (DRP1) antibody (BD Biosciences, 611113, RRID:AB_398423), Human SGPL1 Antibody (R&D systems, AF5535, RRID:AB_2188674), rabbit anti-GAPDH antibody (ab9485, RRID:AB_307275), mouse anti-Actin beta monoclonal antibody (ab6276, RRID:AB_2223210), IRDye® 800CW Goat anti-Mouse IgG (RRID:AB_10793856), IRDye® 800CW Goat anti-Rabbit IgG (RRID:AB_10796098), IRDye® 680RD Goat anti-Mouse IgG (RRID:AB_2651128), IRDye® 680RD Goat anti-Rabbit IgG (RRID:AB_2721181) and IRDye® 680RD Donkey anti-Goat IgG (RRID:AB_10956736). Fluorescent probes - MitoTracker™ Red (M22425, Thermo Fisher Scientific) and pAcGFP1-Mito Vector (632432, Clontech).

### 2.2 Cell lines employed

HeLa control and *SGPL1* knockout cell lines were generously provided by Professor Britta Brügger (Heidelberg University Biochemistry Centre BZH, Germany). S1P lyase disruption in this cell line was achieved by CRISPR-associated RNA-guided endonuclease Cas9 technology by the Brügger group and validated as a system to study protein-sphingolipid interactions [19]. For our study they represent an isogenic *SGPL1*- knockout cell line for investigation. Cells were cultured in Minimum Essential Medium Eagle with Alpha Modification (αMEM, Sigma M8042) supplemented with 10 % FBS, 1% penicillin/streptomycin and 200 mM L-glutamine (Sigma G7513) at 37 °C in 5% CO_2._

### 2.3 Primary fibroblast cell culture

Fibroblast isolation was performed from skin biopsies taken from two patients with loss of function mutations in *SGPL1* (**p.F545del** and p.S65Nfs*11). Immediately after excision, the specimen was incubated in DMEM high glucose supplemented with 10 % Fetal Bovine Serum (FBS) and 1% Penicillin/Streptomycin. Using an aseptic technique, the skin specimen was cut into 1 mm cubes and subsequently digested using a mixture of nutrient media (DMEM high glucose supplemented with 20 % FBS, 1% penicillin/streptomycin and 1:100 non-essential amino acids), 0.25 % collagenase and 0.05 % Dnase I. The mixture was incubated at 37 °C in 5% CO_2_ overnight. The following day, the digestion mixture was centrifuged at 1000 rpm for 5 min and the pellet re-suspended in fibroblast maintenance media (DMEM high glucose with 20 % FBS, 1% penicillin/streptomycin and 1:100 non-essential amino acids). The re-suspended mixture was plated in a 0.1 % gelatin coated T25 flask and left in an incubator at 37 °C in 5% CO_2_ until fibroblast cultures were established.

### 2.4 Measurement of sphingolipid intermediates

Control and patient-derived dermal fibroblasts as well as isogenic HeLa cell lines were cultured as above. Cells were trypsinized, fresh media added and cell counting performed using a hemocytometer. For each cell line, 1.5 × 10^6^ cells were pelleted at 10,000 g for 5 min. Pellets were washed 3x in PBS and stored at −80 °C. Lipidomic analysis was performed by mass spectrometry at the Research Unit on BioActive Molecules (RUBAM), Department of Biomedicinal Chemistry IQAC-CSIC, Barcelona (Spain).

### 2.5 Steroidogenic capacity of cultured human dermal fibroblasts

Normal human and patient dermal fibroblasts were seeded in duplicate in a 24 well plate at a density of 10^5^ cells per well. Cells were incubated for 24h in EpiLife™ media (Thermo-Fisher MEPI500CA) with Defined Growth Supplement (EDGS, Thermo-Fisher S0125). Growth supplemented media was then removed and cells washed three times with PBS. Growth serum free media was added to each well. For stimulation, progesterone 10^−6^M was added only to treatment wells with additional stimulation after 12h. After 24 h, media was removed, centrifuged to remove cell debris and supernatants used for cortisol quantification by ELISA (R&D Systems Cortisol assay parameter kit, KGE008B).

### 2.6 Immunofluorescence staining

For MitoTracker staining of mitochondria, fibroblast and HeLa cells were seeded at a density of 2.5 × 10^3^ per well (24 well plate) on glass coverslips. The MitoTracker lyophilized probe was reconstituted in anhydrous DMSO to a stock concentration of 1 mM. A working concentration of 100 nM was established by dilution in nutrient media prior to addition to cells and incubated at 37 °C in 5% CO_2_ for 30 min. After incubation, cells were washed twice with phosphate buffered saline (PBS) and coverslips fixed with 4% paraformaldehyde for 15 min. Permeabilization was achieved by addition of 0.2 % Triton X-100 for 5 min. Coverslips were counterstained with DAPI and washed with PBS twice prior to mounting on microscope slides. Images were obtained using the 63x oil objective of the confocal Laser scanning microscope 710. Confocal Z-stack images of immuno-stained cells (25 cells per condition) were taken at 1 μm intervals then subjected to volumetric analysis using surface rendered 3D reconstruction by Imaris image analysis software (Imaris 7.6.1 Bitplane, Concord, USA). Morphometric analysis of reconstructed mitochondria was performed using the “ImarisSurface” in-built software tool.

### 2.7 Oxygen consumption rate measurement (Mito Stress Test)

The Seahorse XF Extracellular Flux Analyser (Seahorse Bioscience Inc.) was used to measure the rate of mitochondrial oxidative phosphorylation using a fluorescent biosensor, which detects the oxygen consumption and glycolytic rates of cells seeded in microplates. On the day prior to the assay, cells were seeded in Seahorse XF-96 microplates at specific, previously determined densities (human dermal fibroblasts - 3 × 10^5^ per well and HeLa cells - 3 × 10^4^ per well, with 10–12 replicates per sample) and placed into an incubator overnight (5% CO_2_, 37 °C). The provided sensor cartridge was hydrated with Seahorse XF Calibrant and placed at 37 °C in a non−CO_2_ incubator overnight (or at least 5 h).

On the day of the assay, preparation of assay medium was performed by supplementing Seahorse XF base medium to achieve the following concentration: 10 mM glucose, 1 mM sodium pyruvate, 2 mM l-glutamine (pH of the assay medium was then adjusted to 7.4). The assay medium was placed into a water bath at 37 °C until ready for use. Injection compounds were prepared at the following stock concentrations: Oligomycin (100 μM), FCCP (100 μM) and rotenone/antimycin A (50 μM). Using a starting well volume of 175 μL, each compound was injected into the hydrated sensor plate (Oligomycin – Port A, FCCP – Port B, Rotenone/Antimycin A – Port C) at a volume of 25 μL with varying final well concentrations depending on cell type used. The sensor plate was placed into the analyser for calibration. The Seahorse XF Cell Culture Microplate was removed from the incubator and examined under the microscope to determine cell viability and desired confluence. Cells were supplemented with 175 μL of prepared assay medium and placed into a non−CO_2_ incubator (37 °C) for 45 min to 1 h. After sensor plate calibration, the culture microplate was loaded into the analyser. Basal measurements of oxygen consumption and extracellular acidification rates were taken prior to and following injection of prepared compounds. Data retrieved was normalised to the protein concentration of each well determined by a Bradford assay (Bio-Rad).

### 2.8 RNA isolation and cDNA synthesis

RNA was extracted from cell lines using the **RNeasy mini kit** (Qiagen) according to the manufacturer’s instructions. Genomic DNA removal was performed using an RNase-Free DNase Set (Qiagen, 79254).

For cDNA synthesis, 1 μg of RNA (with 10 mM random hexamer and nuclease free water to a volume of 15 μL) was incubated at 70 °C for 5 min. MuMLV reverse transcriptase enzyme and 10X buffer, Rnase Inhibitor and dNTP were then added to the reaction and placed on a thermo-cycler at 25 °C for 10 min, 42 °C for 90 min and 70 °C for 15 min.

### 2.9 RT-qPCR

qPCR assays were performed using the Kapa SYBR FAST qPCR Kit for analysis of mitochondrial genes *STAR*, *MFN1*, *MFN2* and *DNM1L* expression. Primer sequences were obtained from previously published sources [45, 46, 47] (Supplementary Table 1). A master mix was prepared and adjusted for number of reactions based on the following template: KAPA SYBR FAST qPCR Master Mix (2X) - 5 μL, Forward primer - 0.2 μL (200 nM), Reverse primer - 0.2 μL (200 nM), ROX low - 0.2 μL and Nuclease free water - 3.4 μL. Using a 96-well plate, 20 ng cDNA was added to each well to make a final reaction volume of 10 μL. Thermo-cycling conditions were as follows: DNA denaturation at 95 °C for 3 min followed by 40 cycles of 95 °C for 3 s, 60 °C for 30 s and 72 °C for 10 s. Gene expression levels were calculated using the 2(-Delta Delta C(T)) (ΔΔCT) method with endogenous GAPDH used to normalise data.

### 2.10 Western blotting

Fibroblast and HeLa cell lines were seeded into 6 well plates and whole cell lysates prepared by addition of RIPA buffer (Sigma Aldrich) supplemented with protease and phosphatase inhibitor tablets (Roche). Protein concentrations were quantified using a Bradford protein assay (Bio-Rad). Lysates were either incubated at 37 °C (mitochondrial oxidative phosphorylation complexes using Total OXPHOS Rodent WB Antibody Cocktail) or denatured by addition of Laemmli sample buffer 2X (Sigma Aldrich) and boiled for 3−5 min at 95 °C. 15−20 μg of protein were loaded into wells of a 4–20 % SDS-PAGE gel (Novex) prior to electrophoretic separation using MOPS buffer. Protein transfer to PVDF membrane was achieved by electro-blotting at 15 V for 45 min. The membrane was blocked with 5% fat free milk in PBS/0.1 % Tween-20 and left to gently agitate for 1 h. Primary antibody was added at a concentration of 1:1000 and either anti-GAPDH or β-actin (1:10,000) used as a housekeeping control. Primary antibody incubation was overnight at 4 °C with gentle agitation. The membrane was then washed for 5 min (X3) with Tris Buffered saline-Tween20 (TBST). Secondary anti-mouse (OXPHOS complexes 1-V, DLP1, actin beta), anti-rabbit (PARP, GAPDH) or anti-goat antibodies (SGPL1) were added at a concentration of 1:5000 to blocking buffer and the membrane incubated at 37 °C for 60–90 minutes. The membrane was subsequently washed three times (5 min each) with TBST and visualized with the LI−COR Image Studio software for immune-fluorescent detection.

### 2.11 Statistics

In studies in which statistical analyses were performed, a 2-tailed Student’s *t*-test was used to generate P values. P values less than or equal to 0.05 were considered significant. Data are presented as mean ± SD in all figures in which error bars are shown.

### 2.12 Study approval

This study was approved by the Outer North East London Research Ethics Committee, reference number 09/H0701/12 and London-Chelsea National Research Ethics Service committee, reference number, 13/LO/0224.

## 3 Results

### 3.1 Patient cells and clinical phenotype

**Fig. 2:**
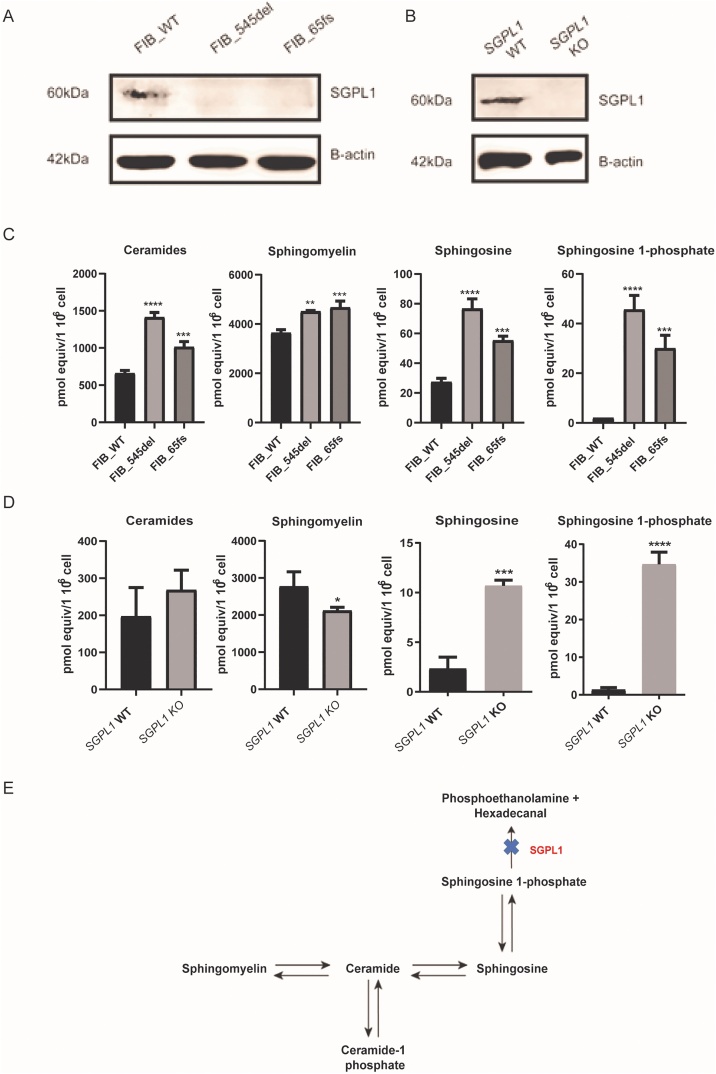
**SGPL1 deficiency leads to accumulation of upstream sphingolipids in patient dermal fibroblasts and the isogenic *SGPL1* knockout HeLa cell line.** A. FIB_WT (control) showed a detectable band at 60 KDa while both patient cell lines were SGPL1 null. B. Similarly, S1P Lyase was expressed in HeLa control lysates and absent in knockout cells. C, D. Levels of sphingolipid intermediates in control and patient-derived dermal fibroblasts as well as isogenic HeLa cell lines. E. S1P lyase deficiency alters the sphingolipid rheostat leading to accumulation of upstream lipids. (*p < 0.05, **p < 0.01, ***p < 0.001, ****p.0001) **Data are presented as the mean of three experiments.**

Sphingosine-1-phosphate lyase insufficiency represents a clinically heterogeneous condition, with the majority of mutations reported in single families and variability in phenotype seen even with the same mutation. Fibroblast cell cultures were established from two affected individuals (published by Prasad et al., 2017) [4] with adrenal failure and steroid resistant nephrotic syndrome (SRNS), **FIB_545del**, from patient 1 with p.F545del (c.1633_1635delTTC, an in-frame deletion) and **FIB_65fs**, from patient 2 with p.S65Nfs*11 (c.261 + 1G > A, a canonical splice-site change), each mutation has only been reported in a single family. These were assayed alongside a control fibroblast cell line (Primary dermal fibroblast, normal human neonatal origin, ATCC® PCS­201 ­ 010™), **FIB_WT**. The two patients express a similar constellation of phenotypic features (PAI, SRNS, ichthyosis, primary hypothyroidism and neurodevelopmental delay). Patient 2 however had earlier onset of disease, of greater severity and more rapidly progressive than patient 1. Lyase activity of the p.F545del mutation has been previously assessed. Hexadecanal production, as a surrogate of lyase activity is almost zero when the in-frame deletion, p.F545del, is expressed in *Sgpl1*^−/−^ mouse embryonic fibroblasts [4]. The single nucleotide substitution c.261 + 1G > A; FIB_65fs, results in aberrant splicing, leading to a frameshift and premature termination codon before the functional domains of the protein (unpublished data). Hence, even if the mRNA message is not removed by nonsense mediated mRNA decay, any resultant protein is unlikely to have lyase activity. Furthermore, in both patient fibroblast cell lines and the *SGPL1* KO HeLa cells, S1P lyase was undetectable by Western blot (Fig. 2 A, B).

### 3.2 Sphingolipid intermediate analysis

Lipidomic analysis performed on control and patient-derived dermal fibroblasts revealed significantly increased levels of ceramides, sphingomyelin, sphingosine and sphingosine 1-phosphate in both patient cell lines (Fig. 2C) whereas, *SGPL1* KO HeLa cells only showed markedly increased levels of the immediate upstream precursors, sphingosine and sphingosine 1-phosphate (Fig. 2D).

### 3.3 Progesterone stimulation of fibroblasts and mRNA expression of *STAR*

**Fig. 3:**
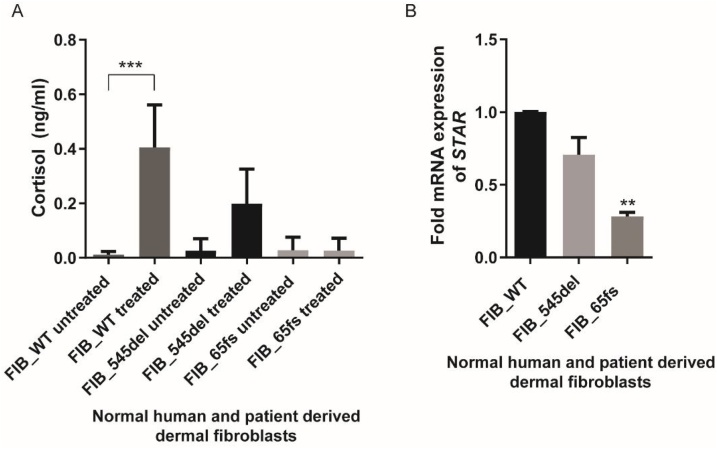
**Progesterone-stimulated cortisol production in dermal fibroblasts and *STAR* mRNA expression.** A. Progesterone treatment produced a reduced cortisol response in patient cell lines in comparison to control fibroblasts. B. *STAR* mRNA expression is significantly reduced in FIB_65Fs dermal fibroblasts (**p < 0.01, ***p < 0.001). **Data are presented as the mean of three experiments.**

The skin can be considered to be a steroidogenic organ and stimulation of dermal fibroblasts using the substrate precursor, progesterone, can induce cortisol accumulation. The steroidogenic capacity of cultured fibroblasts was explored using a protocol outlined by Slominski et al. (2005) [16]. A significant response to progesterone stimulation was noted in FIB_WT human dermal fibroblasts. A measureable, but significantly lower, cortisol response was noted in FIB_545del (p < 0.05) while there was no response in FIB_65fs (Fig. 3A). This would suggest that S1P lyase deficiency can have a significant effect on steroidogenesis downstream of progesterone and its metabolites.

Further upstream, the first step of steroidogenesis is the transport of cholesterol from the outer to the inner mitochondrial membrane by the steroidogenic acute regulatory protein (STAR). Interestingly, we also see reduced expression of *STAR* in FIB_65fs while in FIB_545del, mRNA levels of *STAR* were unaltered (Fig. 3B). In order to assess whether these differential changes correlated to alterations in mitochondrial dynamics and morphology, fluorescence microscopy was undertaken.

### 3.4 Assessment of mitochondrial morphology and volumetric analysis

**Fig. 4:**
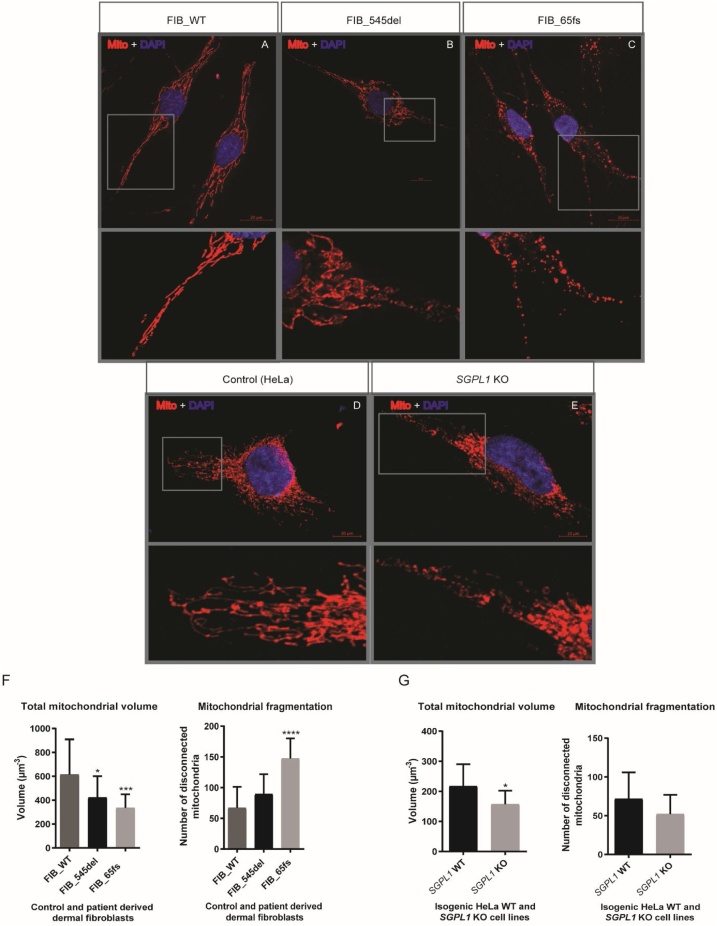
**Confocal microscopic images of immuno-stained cells exhibiting distinct mitochondrial phenotypes.** Panels (A-C) exhibit alterations in mitochondrial architecture in patient fibroblasts compared to FIB_WT. Scale bar = 20 μm. FIB_545del mitochondria are predominantly hyper-fused in contrast to fragmentation seen in FIB_65fs. Panels (D-E) show morphological features of mitochondria in isogenic HeLa cell lines exhibiting mitochondrial hyper-fusion in KO cells. Scale bar = 10 μm. F. Mitochondrial volume was lower in both patient fibroblasts with an increased number of disconnected mitochondrial fragments in FIB_65fs. G. Mitochondrial volume was similarly reduced in *SGPL1*-KO HeLa cells when compared to *SGPL1*-WT. (*p < 0.05, ***p < 0.001, ****p < 0.0001).

Mitochondrial architecture in patient fibroblasts/*SGPL1* knockout HeLa cell lines and their matched controls was examined by confocal microscopy following labelling with the fluorescent dye MitoTracker™ RED (which accumulates in active mitochondria) and DAPI nuclear counterstain. When compared to FIB_WT, FIB_545del mitochondria were hyper-fused and elongated whereas FIB_65fs mitochondria were markedly fragmented. HeLa *SGPL1*-KO cells showed a degree of mitochondrial heterogeneity with a preponderance of fusion events in comparison to *SGPL1-*WT (Fig. 4A – E).

Confocal Z-stack images of stained cells were subjected to volumetric analysis using Imaris image analysis software (Imaris 7.6.1 Bitplane, Concord, USA) to estimate mitochondrial number and volume. Mitochondrial volume was reduced in fibroblasts from both patients compared to FIB_WT: (FIB_545del; 421.7 ± 178.8 μm^−3^) **p < 0.05** and (FIB_65fs; 334.3 ± 114.8 μm^−3^) **p < 0.001** vs. (FIB_WT; 613.8 ± 295 μm^−3^). Additionally, the number of disconnected mitochondria, that is physically separate or discrete mitochondrial structures, representative of mitochondrial fragmentation, per cell was increased in FIB_65fs (FIB_65fs; 147.3 ± 32.83) compared to FIB_WT (66.9 ± 34.21) **p < 0.0001** while there was no significant change in FIB_545del (89.45 ± 32.33). HeLa *SGPL1*-KO cell line also showed a significant reduction in mitochondrial volume **p < 0.01** (156.7 ± 45.63 μm^−3^) vs *SGPL1*-WT (217.1 ± 73.26 μm^−3^) (Fig. 4F, G). However, the number of disconnected mitochondria per cell was unaltered (*SGPL1*-WT; 71.75 ± 34.13) vs (*SGPL1*-KO; 51.9 ± 25.01).

### 3.5 mRNA expression of mitochondrial fusion (*MFN1*/*MFN2*) and fission (*DNM1L*) genes

**Fig. 5:**
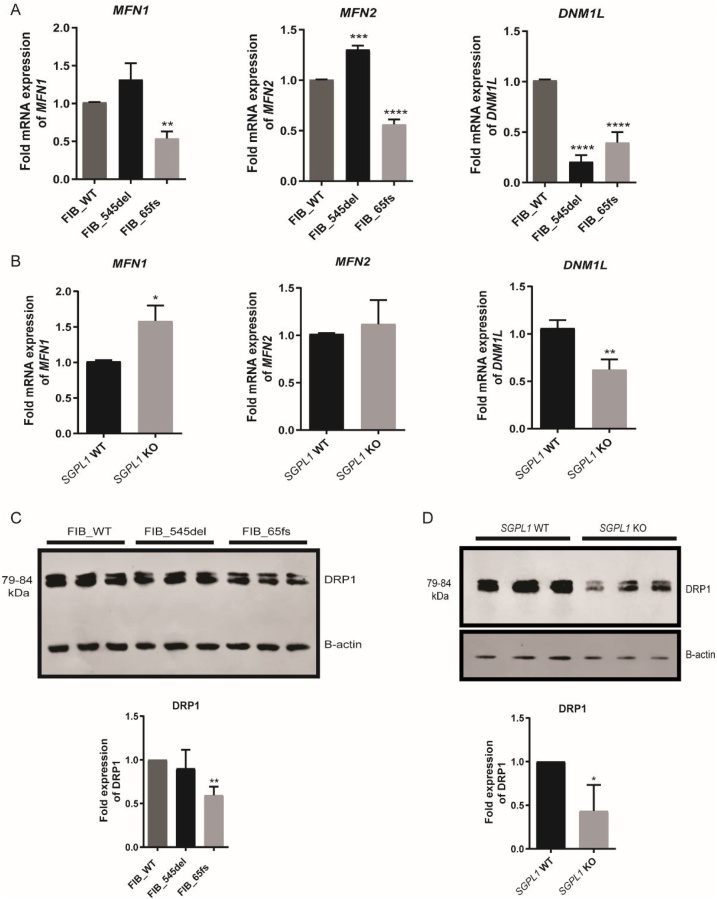
**Fold expression of mitochondrial fusion (*MFN1*, *MFN2*) and fission (*DNM1L*) genes at mRNA and protein level in control and patient fibroblasts and HeLa cell lines.** A. *MFN1/2* levels are reduced in FIB_65fs whereas *MFN2* appears upregulated in FIB_545del while *DNM1L* was downregulated in both patient fibroblasts. B. *MFN1* levels are increased in *SGPL1*-KO cells whereas *DNM1L* (DRP1) appears significantly downregulated. C. Western blot showing levels of DRP1 protein expression with similar reduction seen in FIB_65fs but not FIB_545del. D. DRP1 protein expression with reduction consistent with mRNA levels seen in *SGPL1*-KO HeLa cell lysates. (*p < 0.05, **p < 0.01, ***p < 0.001, ****p < 0.0001) **Data are presented as the mean of three experiments.**

Mitochondrial fission and fusion modulate dynamic mitochondrial morphological transitions. In order to probe whether altered transcript levels of fusion genes, Mitofusins 1 and 2 (*MFN1*, *MFN2*) and fission gene *DNM1L* (encodes the GTPase dynamin-1-like protein, DRP1) were linked to the distinctive mitochondrial phenotypes seen, RT-qPCR was performed and normalised to housekeeping gene, *GAPDH*. *MFN2* expression was upregulated in FIB_545del (p < 0.0001) while the opposite was seen in FIB_65fs (p < 0.0001). *MFN1* expression was not significantly altered in FIB_545del, whilst it was reduced in FIB_65fs compared to control (p < 0.01). However, *DNM1L* was downregulated in both patient cells (p < 0.0001) (Fig. 5A). Levels of DRP1 were further probed by western blotting of fibroblast cell lysates. Reduced levels in FIB_65fs cell lysates were consistent with RT-qPCR data however in FIB_545del, protein levels were unaltered in contrast to mRNA levels (*DNM1L*) (Fig. 5C). *SGPL1*-KO HeLa cells showed an upregulation of *MFN1* (p < 0.05) and reduced expression of *DNM1L* (p < 0.01) (Fig. 5B). Levels of DRP1 were similarly reduced in *SGPL1*-KO HeLa cell lysates (Fig. 5D).

### 3.6 Expression of mitochondrial oxidative phosphorylation complexes at protein level

**Fig. 6:**
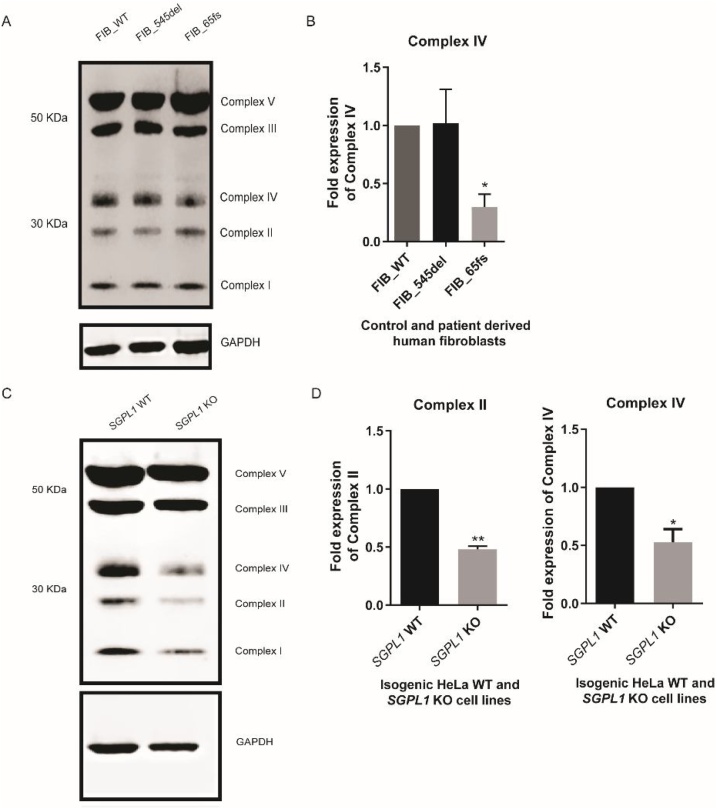
**Western blot of mitochondrial oxidative complexes in patient fibroblasts and HeLa cell lines.** A. Representative western blot of fibroblast cell lines B. Complex IV protein expression appears reduced in FIB_65fs when normalised to GAPDH, while expression levels of respiratory chain proteins in FIB_545del are unaltered. C. Representative western blot of HeLa cell lines D. Complexes II and IV appear reduced in *SGPL1*-KO cell lysates. (*p < 0.05, **p < 0.01) **Data in graphs are presented as the mean of three experiments.**

With mitochondrial morphology disrupted in S1P lyase deficient cells we went on to investigate mitochondrial function in these cell lines. Mitochondrial oxidative phosphorylation is responsible for cellular ATP synthesis and is dependent on the stable organisation of electron transport chain complexes I–V. Cell lysates of control and patient dermal fibroblasts were probed with an antibody cocktail directed against respiratory chain complexes I–V. Although protein levels of each complex in FIB_545del were unaltered compared to FIB_WT, complex IV was significantly downregulated in FIB_65fs (Fig. 6A, B). Expression levels of respiratory chain complexes were reduced in *SGPL1*-KO HeLa cell lysates compared to *SGPL1*-WT with significant downregulation of complexes II and IV (Fig. 6C, D).

### 3.7 Oxygen consumption rate measurement (Mito Stress Test)/Bioenergetic profiling

**Fig. 7:**
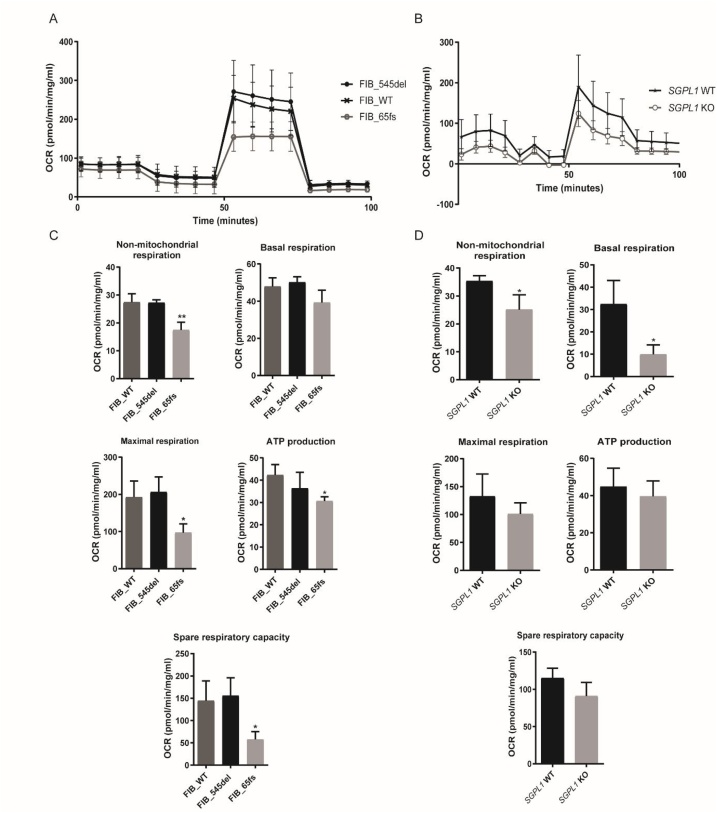
**Respiratory flux profiles of control and patient-derived human dermal fibroblasts and HeLa cell lines.** A, B. Oxygen consumption rates (OCR) of control and patient fibroblasts and HeLa cell lines following injection of optimal concentrations of Oligomycin (4μM), FCCP (1μM) and Rotenone/antimycin A (1μM). Panel C shows decreased respiratory potential of FIB_65fs compared to FIB_WT with significant reductions in non-mitochondrial respiration, maximal respiration, ATP production and spare respiratory capacity. D. Oxygen consumption rates (OCR) of *SGPL1*-WT and *SGPL1*-KO cell lines show decreased respiratory potential of KO cells compared to control with significant reductions in non-mitochondrial and basal respiration. (* p < 0.05, ** p < 0.01); **Data are presented as the mean of three experiments.**

The rate of mitochondrial oxidative phosphorylation in control and patient-derived human dermal fibroblasts as well as HeLa cell lines was measured using the Seahorse XF Extracellular Flux Analyser (Seahorse Bioscience Inc.) after injection of specific modulatory compounds of the electron transport chain. Oligomycin inhibits oxidative phosphorylation and is used to determine mitochondrial ATP production under basal conditions while carbonyl cyanide-4 (trifluoromethoxy) phenylhydrazone (FCCP), a protonophore, abolishes the pH gradient across the inner mitochondrial membrane and gives an estimate of maximal respiration. Rotenone/antimycin A completely suppress mitochondrial respiration even in the presence of potent protonophores such as FCCP and therefore give an estimate of non-mitochondrial respiration. In both FIB_WT and FIB_545del fibroblasts, the mitochondria were respiratory competent, showing no significant difference in parameters of oxidative phosphorylation (Fig. 7A) in FIB_545del compared to FIB_WT; non-mitochondrial respiration, basal respiration, maximal respiration, ATP and spare respiratory capacity (Fig. 7C, Table 1).

**Table 1:** Statistical comparison of measures of oxidative phosphorylation across fibroblast cell lines.

Parameter	FIB_WT (pmol/min/mg/mL)	FIB_545del (pmol/min/mg/mL)	FIB_65fs (pmol/min/mg/mL)	Significance FIB_WT vs. FIB_545del	Significance FIB_WT vs. FIB_65fs
Non-mitochondrial respiration	27.4 ± 3.059	27.3 ± 1.049	17.42 ± 2.818	ns	p < 0.01
Basal respiration	47.87 ± 4.727	50.15 ± 2.969	39.22 ± 6.709	ns	Ns
Maximal respiration	192.3 ± 43.68	205.9 ± 40.71	96.83 ± 23.8	ns	p < 0.05
ATP production	42.27 ± 4.723	36.3 ± 7.208	30.62 ± 1.91	ns	p < 0.05
Spare respiratory capacity	144.4 ± 44.41	155.7 ± 40.04	57.61 ± 17.59	ns	p < 0.05

However, FIB_65fs showed significant reductions in non-mitochondrial respiration maximal respiration, ATP production and spare respiratory capacity. Mitochondrial oxidative capacity appeared to be higher in HeLa control cells (Fig. 7B) with lower non-mitochondrial and basal respiration seen in *SGPL1* knockout cells. Although a similar trend was seen for maximal respiration, ATP production and spare respiratory capacity in the knockout cell line, this was not statistically significant (Fig. 7D, Table 2).

**Table 2:** Statistical comparison of measures of oxidative phosphorylation between *SGPL1*-WT and *SGPL1* KO HeLa cell lines.

Parameter	*SGPL1*-WT (pmol/min/mg/mL)	*SGPL1* KO (pmol/min/mg/mL)	Significance *SGPL1*-WT vs. *SGPL1* KO
Non-mitochondrial respiration	35.32 ± 1.935	25.09 ± 5.343	p < 0.05
Basal respiration	32.41 ± 10.54	9.903 ± 4.177	p < 0.05
Maximal respiration	132.5 ± 40.05	100.8 ± 20.03	ns
ATP production	44.81 ± 9.929	39.54 ± 8.336	ns
Spare respiratory capacity	115.1 ± 13.14	90.86 ± 18.33	ns

### 3.8 Expression of cleaved PARP1, poly (ADP-ribose) polymerase, as a marker of apoptosis

Ceramide has been linked to mitochondrial apoptosis via induction of mitochondrial outer membrane permeabilization (MOMP), release of inter-membranal cytochrome c into the cytosol and activation of executioner caspases. Following release of cytochrome c, cells can exhibit several features of programmed cell death including cleavage of PARP, poly (ADP-ribose) polymerase. Sphingosine can also induce cell death independent of ceramide, whilst S1P is regarded as an anti-apoptotic molecule. Thus, the balance of sphingolipid metabolism can determine cell fate [20].

**Fig. 8:**
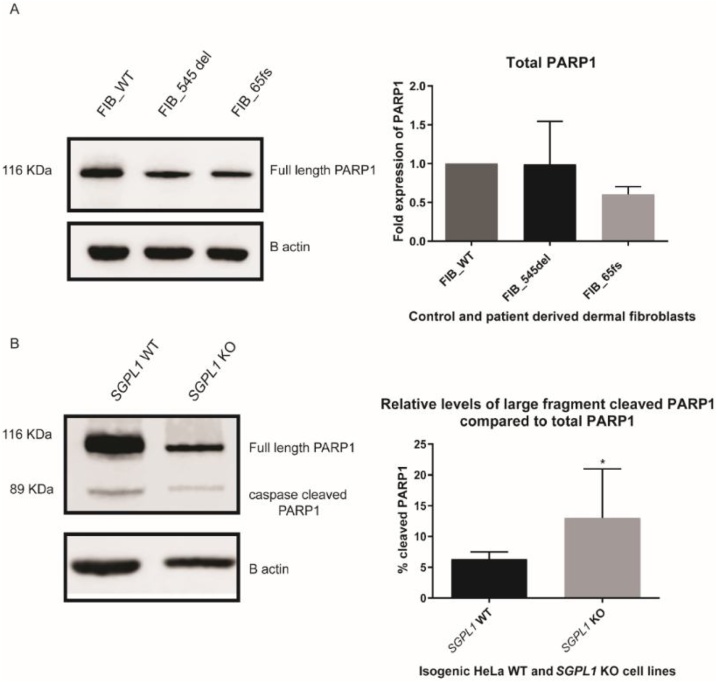
Western Blotting for endogenous full length *PARP1* and cleaved *PARP1*. **A**. Unaltered levels of total PARP1 between control and patient fibroblast cell lines and absence of cleaved *PARP1*. **B**. *SGPL1*-KO HeLa cell lysates showed an increased percentage of caspase-3 cleaved PARP1. (*p < 0.05) **Data are presented as the mean of three experiments.**

Western blotting for endogenous full length PARP1 (116 KDa) and 89 kDa cleaved PARP1 was undertaken in HeLa and fibroblast cell lysates to determine the effect of loss of *SGPL1* on apoptosis. In *SGPL1*-KO cells, there was an increased ratio of cleaved to total PARP1 compared to *SGPL1*-WT suggesting increased caspase-3 mediated apoptosis (Fig. 8). Total endogenous PARP1 levels were similar between FIB_WT and patient fibroblast cell lines however there was no detectable cleaved PARP1 in fibroblast cell lines. This correlates with previous work suggesting apoptotic resistance in both quiescent and senescent fibroblasts (Hampel et al., 2005).

## 4 Discussion

Sphingosine-1-phosphate lyase insufficiency comprises multi-systemic and progressive disease. Steroid resistant nephrotic syndrome and neurological disease are described in other disorders of sphingolipid metabolism, however, in contrast to the other sphingolipidoses, S1P lyase deficiency is associated with multiple endocrinopathy. The constellation of adrenal, gonadal and thyroid disease has only been described in one other non-autoimmune inherited form of adrenal insufficiency, secondary to mutations in *NNT,* encoding nicotinamide nucleotide transhydrogenase that is involved in redox potential transfer in the mitochondrial respiratory chain [21]. Mitochondrial disorders can be difficult to recognise due to their multi-systemic presentation and effect. Primary mitochondrial disease and adrenal insufficiency have only been characterised in a small number of patients with underlying pathology linked to defective ATP production and leading to reduced adrenal steroid metabolism [[22], [23], [24], [25]].

Loss of the highly degradative enzyme sphingosine 1-phosphate lyase can potentiate accumulation of sphingolipid intermediates leading to disrupted homeostasis. Mass spectrometry of sphingolipids in fibroblasts derived from the two patients showed elevation of several lipid intermediates. Both FIB_545del and FIB_65fs had increased levels of all sphingolipid intermediates (S1P, sphingosine and ceramide species), together with an increase in sphingomyelin. Elevations in ceramide levels within mitochondria can cause inner mitochondrial membrane dysfunction [26].

The steroidogenic capacity of dermal fibroblasts is well established with previous work highlighting the expression of *CYP11A1*, *CYP17A1* and *CYP21A2* in skin [17,18,27,28]. Progesterone stimulation of control and patient fibroblasts elicited a significant cortisol response in FIB_WT, with a measurable, albeit significantly lower, response in FIB_545del and very low basal and stimulated cortisol levels in FIB_65fs. Sphingolipid intermediates, ceramide and sphingosine are implicated in attenuating steroidogenesis whilst S1P potentiates steroid production [14,15]. In both patient fibroblasts a significant increase in all 3 of these intermediates is seen. This would suggest that ceramide and sphingosine effects may outweigh those of S1P, in these S1P lyase deficient cells. The effects on S1P signalling also need to be further explored, as S1P extracellular receptor internalisation and attenuation has been described in the face of overwhelming S1P [[29], [30], [31]].

Sphingolipid accumulation can impair mitochondrial morphology and function, which could impair steroidogenesis. The marked lack of steroid production particularly in the FIB_65fs cell line was concomitant with a severe mitochondrial phenotype, with much reduced fusion and increased mitochondrial fragmentation. In FIB_65fs cells, there was a significant reduction in mitochondrial metabolism with lowered oxidative capacity and mitochondrial mass when compared to FIB_WT. The mitochondria in these fibroblasts were markedly fragmented in keeping with the downregulated mRNA levels of both fusogenic genes, *MFN1* and *MFN2.* Mitochondrial fusion is essential for steroidogenesis hence alterations in fusion rates may hamper steroid production [32]. Reduced mRNA expression of *STAR*, responsible for the transport of cholesterol across the mitochondrial membrane at the start of steroidogenesis, was observed in FIB_65fs cells, in keeping with previous studies linking abrogation of *Mfn2* with reduced *Star* mRNA expression [33]. Interestingly, mRNA and protein expression of *DNM1L*/DRP1 were also reduced, suggesting an increase in DRP1-independent fragmentation in FIB_65fs. Downregulation of *DNM1L* in mammalian cells has previously been associated with reduced mitochondrial respiration and cellular ATP levels due to a lack of fission [34,35].

Whilst total mitochondrial volume was also reduced in FIB_545del cells, they showed unaltered respiratory chain function and normal expression of electron transport chain complexes. This may represent a compensatory adaptive response due to mitochondrial ultrastructural changes in these fibroblasts suggestive of increased fusion with overexpression of *MFN2* and reduced mRNA levels of *DNM1L* [[36], [37], [38]]. *MFN2* to an extent regulates nuclear expression of oxidative phosphorylation complexes and subsequently energization of the respiratory chain [36]. Inhibition of mitochondrial fusion leads to disruption of the electron transport chain, with *MFN2* depletion resulting in reduced expression of respiratory chain complexes I, II, III and V and decreased oxygen consumption and glucose oxidation. Conversely, *MFN2* overexpression leads to enhancement of respiratory chain function [36]. Previous work has established a partial redundancy in function between MFN1/2 in that overexpression of either protein is able to compensate for absence of the other, thereby promoting mitochondrial fusion [36,39,40]. Increased expression of *MFN2* in FIB_545del was associated with preserved *STAR* mRNA levels.

Structural reorganization and altered expression of respiratory chain complexes have been linked to dysregulated mitochondrial dynamics. Protein expression of complexes I–V in FIB_545del was unaltered compared to wild type. In FIB_65fs cells, where both mRNA and protein expression of *DNM1L*/DRP1 are reduced, the level of Complex IV (cytochrome c oxidase) is significantly reduced. Silencing of *DNM1L* in mammalian cells has been linked to abrogation of complex IV activity [35]. Furthermore, complex IV deficiency has been linked to mitochondrial fragmentation and may account for the mitochondrial phenotype of FIB_65fs [41]. Complex IV (COX) is a 200 KDa inner mitochondrial membrane complex that forms the terminal enzyme of the respiratory chain. COX deficiency is often associated with mitochondrial disease and potentiates increases in oxidative stress mediated apoptosis [42]. In vitro, several sphingolipid intermediates (C16-ceramide, sphingosine and sphinganine) have been shown to acts as inhibitors of complex IV function in mitochondrial isolates [43].

Overall, the findings demonstrate a more severe impact on mitochondrial morphology and function in the FIB_65fs line and a lesser effect that appeared to be compensated in the FIB_545del line. Despite similarly dysregulated sphingolipid metabolism in both patients, their differing mitochondrial phenotypes could also be due to different genetic or environmental modifiers. Sphingosine-1-phosphate lyase insufficiency is clinically heterogeneous, with differences in phenotype seen even amongst members of the same kindred [4,5]. Of note, patient 2, from whom FIB_65fs fibroblasts were derived, displayed a more severe clinical phenotype of earlier onset compared to patient 1.

A pathological effect of disrupted sphingolipid metabolism on mitochondrial dynamics was supported by the study of an available isogenic *SGPL1*-KO cell line. *SGPL1* KO HeLa cells showed increased levels of sphingosine and sphingosine 1-phosphate but this cumulative effect was lost higher upstream of the metabolic defect. When compared to control, mitochondria in *SGPL1* KO HeLa cells, like the FIB_545del, appeared hyper-fused in keeping with increased *MFN1* mRNA levels and downregulation of *DNM1L.* Inhibition of DRP1 mediated fission in HeLa cells can lead to loss of mitochondrial DNA, uncoupled cellular respiration and increased autophagy in *DNM1L* knockdown cells [29]. In our isogenic *SGPL1*-KO HeLa cell lines we observe reduced basal or endogenous respiration with reduced expression of respiratory chain complexes II and IV suggesting that loss of DRP1 is linked to inefficient oxidative phosphorylation.

Modulated complex IV activity has been linked to increased apoptosis. Poly (ADP-ribose) polymerase 1 (PARP1) is a ubiquitous nuclear protein involved in DNA repair that acts as a substrate for caspase-3 with caspase mediated cleavage of endogenous PARP1 being a recognised marker of apoptosis. Control and *SGPL1* KO HeLa lysates, demonstrated an increased ratio of cleaved to total PARP1 in KO cells compared to control, suggestive of increased cell death. Of interest, ceramide levels were not significantly different in comparison with controls in this cell line. Sphingosine, however, which was significantly increased, has been shown to induce programmed cell death independent of ceramide effects [44]. These include cytochrome c release, activation of caspase 3 and PARP1 cleavage, amongst other effects. The overall effect of sphingolipid accumulation on cell viability needs to be further assessed, with organelle disruption likely to be cell/ tissue- specific. It would also be of interest to interrogate the effect of a reduction of product downstream of S1P lyase, for instance phosphoethanolamine used in the synthesis of phosphatidylethanolamine (PE) which forms a major component of the inner mitochondrial membrane. Whilst S1P degradation by S1P lyase does not constitute a major pathway for *de novo* PE synthesis, S1P lyase deficiency may also affect mitochondrial function by this route.

Further analysis of mitochondrial fusion/ fission events and how they affect the integrity of mitochondria and mitochondrial homeostasis in S1P lyase deficiency is required and may represent a target for therapeutics. Steroidogenesis requires modulation of mitochondria-mitochondria interactions and mitochondrial and endoplasmic reticulum interaction at the mitochondrial associated membrane interface. Characterising the effect on the endoplasmic reticulum would also therefore be pertinent in the study of the pathogenic mechanism of this disease. Future work involves investigation of the interplay of disrupted mitochondrial dynamics and sphingolipid intermediate signalling on acute steroidogenesis in an isogenic adrenocortical cell model, H295R, with *SGPL1* KO developed using CRISPR/Cas9 technology.

## 5 Conclusions

*SGPL1* deficiency results in a mitochondrial disorder. Aberrant sphingolipid metabolism including sphingosine and ceramide generation compromises mitochondrial integrity leading to reduced mitochondrial function. Variability in mitochondrial phenotype between the two patient cell lines may be due to differing genetic backgrounds and/or involvement of other components of the mitochondrial pathway independent of the *SGPL1* deficiency. The complex interplay between background genetics, sphingolipid biosynthetic defects, mitochondrial dynamics and steroidogenesis is key to understanding the phenotypic expression of this disease.

## Funding

This work was supported by the Medical Research Council UK(MR/K020455/1 to LAM), Barts and the London Charity (MGU0361, 2017 to LAM); 10.13039/501100007155Medical Research Council (MRC) UK Clinical Academic Research Partner Grant (MR/T02402X/1, 2019 to RP), Medical Research Council (MRC) UK/ Academy of Medical Sciences Fellowship Grant G0802796 (to LFC).

## references

[[45], [46], [47]].

## CRediT authorship contribution statement

**A. Maharaj:** Investigation, Formal analysis, Writing - original draft. **J. Williams:** Investigation, Formal analysis, Writing - review & editing. **T. Bradshaw:** Methodology. **T. Güran:** Investigation, Writing - review & editing. **D. Braslavsky:** Investigation, Writing - review & editing. **J. Casas:** Investigation, Formal analysis, Writing - review & editing. **L.F. Chan:** Conceptualization, Supervision, Writing - review & editing. **L.A. Metherell:** Conceptualization, Supervision, Formal analysis, Writing - original draft. **R. Prasad:** Conceptualization, Supervision, Formal analysis, Writing - original draft.
