# Supplementary material for: Sphingosine-1-phosphate lyase (SGPL1) deficiency is associated with mitochondrial dysfunction
Source: J Steroid Biochem Mol Biol. 2020 Sep;202:105730. doi: 10.1016/j.jsbmb.2020.105730 (PMC7482430; doi:10.1016/j.jsbmb.2020.105730)
Supplement: Supplementary file 1 [file mmc1.docx]

**Supplementary information:**

**Table 1. Primer sequences used for RT-qPCR**

| GENE | Primer sequence | Source |
| --- | --- | --- |
| STAR | Sense – AAGAGGGCTGGAAGAAGGAG, Antisense – TCTCCTTGACATTGGGGTTC | Ruiz-Babot *et. al* ^30^ |
| MFN1 | Sense – TGTTTTGGTCGCAAACTCTG, Antisense – CTGTCTGCGTACGTCTTCCA | Cartoni *et. al* ^31^ |
| MFN2 | Sense – ATGCATCCCCACTTAAGCAC, Antisense – CCAGAGGGCAGAACTTTGTC | Cartoni *et. al* ^31^ |
| DNM1L | Sense – GTTCCACGCCAACAGAATAC, Antisense - CCTAACCCCCTGAATGAAGT | Romanello *et. al* ^32^ |
